# Knowledge and awareness level of healthcare professional candidate students on inherited metabolic diseases: a cross-sectional study

**DOI:** 10.1186/s12909-023-04548-y

**Published:** 2023-08-09

**Authors:** Nevra Koç, Tuğba Küçükkasap Cömert

**Affiliations:** Department of Nutrition and Dietetics, Gülhane Faculty of Health Sciences, University of Health Sciences, Ankara, Turkey

**Keywords:** Inborn metabolic diseases, Healthcare students, Knowledge, Awareness

## Abstract

**Background:**

Healthcare professionals play a key role in the diagnosis, treatment, and follow-up of inborn metabolic diseases. However, the level of inborn metabolic disease knowledge of prospective healthcare professional students in our country has not yet been determined. Therefore, this study aimed to evaluate the level of knowledge of healthcare professional candidate students about inborn metabolic diseases.

**Methods:**

The knowledge levels of 761 students enrolled in the Department of Nutrition and Dietetics, Child Development, Midwifery, Occupational Therapy, Audiology, Health Management and Social Work at Gülhane Faculty of Health Sciences, Health Sciences University, were evaluated through a questionnaire using a face-to-face interview technique. Correct answers to the questions measuring the level of knowledge were scored as “1”, and incorrect answers were scored as “0”.

**Results:**

The mean knowledge-level score of the individuals was 14.23 ± 4.56. A total of 56.0% of individuals had heard about inborn metabolic diseases before, 37.8% had heard of rare disease organizations/platforms before, and 16.8% had encountered an awareness campaign about inborn metabolic diseases. The level of exposure to awareness-raising campaigns, department of education, and grade level were shown to be factors affecting knowledge levels.

**Conclusion:**

It is necessary to improve the awareness and knowledge levels of health professional candidates involved in the treatment of inborn metabolic diseases. Education curricula in health sciences faculties should be evaluated with this aspect.

## Background

Inborn metabolic diseases (IMDs) Morava et al. have defined it as hereditary disorders, in which impairment of specific enzymes or biochemical pathways is intrinsic to the pathomechanism [1, 2].

There are more than 1400 identified IMDs (phenylketonuria, tyrosinemia, maple syrup urine disease, organic acidemia, homocystinuria etc.) and they are typically inherited as autosomal recessive [3–5]. Almost all of these are rare disorders, but when taken as a whole, the prevalence is 43.4–584 per 100,000 live births throughout the world [6]. Its incidence varies depending on ethnicity, diagnostic tests used, newborn screening programs, technological advancements, reporting rates, awareness, and in particular, the frequency of consanguineous marriages (because of the expression of autosomal recessive gene mutations inherited from a comman ancestor) in society [7].

It has been reported that Saudi Arabia, Egypt, Libya, Bahrain, Tunisia, and India have the highest rates of consanguineous marriage in the world, with rates of 92%, 88%, 86%, 84%, 81%, and 73%, respectively. Consanguineous marriages are common in Turkey (25% in general and 42% in some geographical regions), and in relation to this, it is among the countries with a high incidence of phenylketonuria with IMD with an incidence rate of 1:5049. as well as the fact that metabolic diseases are becoming an increasing public health problem [8].

In some inborn metabolic diseases, many complications (growth and development retardation, ataxi, seizures, hypotonia, leukodystrophy, autism, hepatomegaly, cataracts, dysmorphism) that may arise can be prevented by early diagnosis so that their quality of life can be increased, and some of them can be treated [9]. For this purpose, newborn screening programs are carried out, and genetic counseling services are provided in our country [10]. Raising social awareness, determining the level of knowledge of healthcare professional candidate students who will face the IMD group in the field, identifying deficiencies, and reconsidering the course curricula are recommended measures to be taken [11].

IMD are rare causes of morbidity and mortality in general, but they are important in the sense that they are mostly preventable, and therefore require early diagnosis and treatment [12]. According to data provided by the United Nations Development Program (UNDP), there are 23.529 annual deaths related to IMDs, which account for 0.4% of all child deaths worldwide [13].

It has been determined that studies have been conducted in Malaysia [11], Saudi Arabia [14], and Poland [15] to evaluate IMD knowledge levels in healthcare sciences students, but such a study has not been conducted in Turkey. Although IMDs are common in Turkey, it is known that the courses on IMDs in the health sciences education curriculum of universities that train health professionals are inadequate. In the curricula of 28% of the departments, there are courses about IMDs (2–10 h). There are no one-to-one courses for inborn metabolic diseases, except for the curriculum of the Nutrition and Dietetics Departments in the current core education program in the health sciences undergraduate program. However, these diseases are treated by large teams that include many health professionals. For this reason, it is very important for the health professional in the team to increase their knowledge and skills about IMD in line with their role. 20% of the diagnosis and treatment centers for IMD in Turkey are located in Ankara (25 centers in Turkey, 5 centers in Ankara). Since our faculty is located in Ankara, it causes our students to encounter this patient group frequently. From this perspective, this study was planned and conducted to measure the knowledge and awareness level of University of Health Sciences Gülhane Faculty of Health Sciences students and to evaluate the factors affecting their level of knowledge, determine the situation and make new plans in curriculum.

## Methods

This study was conducted on students enrolled in various departments (Nutrition and Dietetics, Child Development, Midwifery, Occupational Therapy, Audiology, Health Management, and Social Work) at Gülhane Faculty of Health Sciences, University of Health Sciences between 07/04/2022-20/06/2022.

### Ethical considerations

Ethics Committee approval of the study was obtained with the meeting decision of the University of Health Sciences, Gülhane Scientific Research Ethics Committee, dated 26.05.2022, and project number 2022/156, numbered 2022/06. All individuals were informed about the study and signed a written informed consent form. All procedures in the study were carried out following the Declaration of Helsinki.

Questionnaire forms were distributed to 800 students studying at the faculty of health sciences; 761 students agreed to participate in the study. All individuals who were students of the faculty of health sciences were included in the sample. Individuals with a diagnosis of IMDs were excluded from the sample.

There is no standardized tool that measures the level of knowledge of health professionals on this subject. The questionnaire in the study was created by the authors who have clinical studies in the field of inborn metabolic diseases before starting the study and then presented to the opinion of an expert consisting of 1 pediatric metabolism physician, and the literature (11,14,15). The questions were revised in accordance with the opinions. Afterwards, it was applied to the pilot student group.

The data regarding the research were collected using a questionnaire form through the face-to-face interview method. The questionnaire consists of two parts: the first part consists of general information (7 questions) and questions assessing the level of awareness about IMDs (5 questions), and the second part consists of questions assessing the level of knowledge about diagnosis/screening methods (11 questions), treatment (11 questions) and follow-up (5 questions) of IMDs.

Correct answers to the questions measuring the level of knowledge of IMD were scored as “1” and incorrect answers were scored as “0”, and the total score and the score consisting of diagnosis/screening methods, treatment, and follow-up subgroups were calculated for each individual. The total score that can be obtained ranges between 0 and 27.

### Statistical analysis of data

Descriptive analyses were expressed as number (N), percentage (%), mean, and standard deviation (SD) and analyzed with the SPSS Statistics 22.0 software [IBM CORB., Armonk, NY, USA]. Normality analysis was performed for the variables, and it was assumed that they showed normal distribution since the skewness (0.517) and kurtosis (-1.738) values were between + 2.0 and − 2.0 [16]. Independent t test was used to determine whether or not there is a statistically significant difference between the means of the knowledge-level score for screening/diagnosis of IMD, the knowledge-level score for treatment of IMD, the knowledge-level score for follow-up, and the total knowledge score for IMD according to the consanguineous marriage, encountering awareness-raising campaigns about IMD, presence of the subject of IMD in their curriculum.

One Way Anova was used whether or not there is a statistically significant difference between the means of the knowledge-level score for screening/diagnosis of IMD, the knowledge-level score for treatment of IMD, the knowledge-level score for follow-up, and the total knowledge score for IMD according to the department and grade of education. The results were considered significant at p < 0.05.

## Results

The study was conducted on 761 individuals studying at the Gülhane Faculty of Health Sciences, University of Health Sciences. The mean age was 21.20 ± 1.05 years. Of the individuals who participated in the study, 17.6% were students of Child Development, 15.4% of Occupational Therapy, 15.4% of Nutrition and Dietetics, 14.9% of Social Work, 11.9% of Midwifery, 12.4% of Health Management and 10.0% of Audiology Departments. Of these, 13.4% were in 1th grade, 45.1% were in 2nd grade, 32.5% were in 3rd grade, and 8.9% were in 4th grade (Table 1).

**Table 1:** Sociodemographic characteristics of healthcare professional students (n:761)

Variable	N	%
**Age**		
18–21	553	72.7
22–25	203	26.7
> 26	5	0.6
**Department**		
Nutrition and Dietetics	114	14.9
Child Development	133	17.6
Midwifery	90	11.9
Occupational Therapy	117	15.4
Audiology	90	11.9
Health Management	103	13.4
Social Work	114	14.9
**Grade of education**		
1	102	13.4
2	343	45.1
3	247	32.5
4	69	8.9
**Consanguineous marriages in their families**		
Yes	122	16.1
No	639	83.4

Awareness status of healthcare professional students about inborn metabolic diseases was given in Table 2. A total of 56.0% of individuals had heard about IMDs before, 37.8% had heard of rare disease organizations/platforms before, and 16.8% had encountered an awareness campaign about IMDs. Participants were asked whether they knew the names of inborn metabolic diseases in the list given to them. It was asked as multiple choice in the questionnaire form. Among those who stated that they had heard about inborn metabolic diseases before, 47.9% heard about them through social media, 34.1% through TV, 9.7% through hospitals, and 8.3% through family/friends. The IMDs that the participants thought that were the most common in the population were phenylketonuria, galactosemia, and glycogen storage diseases (43.3%, 30.6%, and 26.1%), while the least common were homocystinuria, organic acidemia, and methylmalonic acidemia (8.8%, 7.7%, and 4.1%), respectively. Of the individuals (37.8%) who stated that they had heard of rare disease organizations/platforms before, 33.6% had heard of Spinal Muscular Atrophy (SMA), 6.3% had heard of Rare Diseases Network, 3.2% had heard of Orphanet and PKU Family, 2.8% had heard of the European Union Committee of Experts on Rare Diseases (EUCERD), and 2.5% had heard of the Metabolic Diseases Foundation (METVAK) (Table 2).

**Table 2:** Awareness of healthcare professional students about inborn metabolic diseases (n:761)

Variable	N	%
**Hearing about IMD**		
Yes	426	56.0
No	335	44.0
**Information resources on IMD**		
TV	145	34.1
Social media	204	47.9
Hospital	41	9.7
Family/friends	36	8.3
**Hearing of rare disease organizations/platforms**		
Yes	287	37.8
No	474	62.2
**Rare disease organizations/platforms heard**		
SMA	251	33.6
Rare Diseases Network	48	6.3
Orphanet	24	3.2
PKU Family	24	3.2
EUCERD	21	2.8
METVAK	19	2.5
**Encountered an awareness campaign about IMDs**		
Yes	127	16.8
No	634	83.2

The distribution of healthcare professional students by their correct responses to the questions measuring their level of knowledge about inborn metabolic diseases is shown in Table 3. Regarding screening/diagnostic methods, the statements that genetic screening tests are risky to be applied to healthy pregnant women (18.4%) and genetic screening tests are against religion and beliefs (12.1%) were stated correctly at the lowest level. Regarding treatment, it was found that the sentences “Patients with inborn metabolic diseases cannot breastfeed” (16.7%) and “Patients with inborn metabolic diseases should not be given vitamin/mineral supplements” (17.4%) were answered correctly at the lowest level. Regarding the follow-up, the sentences “There is no need for clinical follow-up after diagnosis of inborn metabolic diseases” (15.4%) and “Clinical follow-up of inborn metabolic diseases is performed by Family Health Centers” (40.1%) were answered correctly at the lowest level (Table 3).

**Table 3:** Distribution of healthcare professional students by their correct answers to questions measuring their level of knowledge about IMD (n: 761)

Variable	N	%
**Screening/Diagnostic Methods**		
Genetic screening tests can be performed on all newborns	563	74.0
Genetic screening tests can be performed on all pregnant women.	488	65.7
It may be risky to perform a genetic screening test on a healthy pregnant woman.	140	18.4
Genetic screening tests are performed by Family Health Centers.	339	44.6
Newborns can be tested for metabolic diseases with a heel stick.	588	79.1
Parents with inborn metabolic diseases can have healthy children.	340	45.8
Healthy parents can have children with inborn metabolic diseases.	530	71.3
Individuals who are carriers of an inborn metabolic diseases show signs of the disease.	380	51.1
For a disease to be defined as hereditary, it must occur in more than one person in the family.	417	54.8
Inborn metabolic diseases also occur in adolescence/adulthood.	311	40.9
It is against religion and belief to conduct genetic screening tests.	91	12.0
**Treatment**		
Inborn metabolic diseases can be treated.	537	70.6
Inborn metabolic diseases can be cured after treatment.	178	23.5
Treatment of inborn metabolic diseases begins immediately after birth.	500	67.3
Individuals with inborn metabolic diseases cannot breastfeed.	127	16.7
Treatment of inborn metabolic diseases lasts a lifetime.	453	61.0
Obesity may occurs in the treatment of patients with inborn metabolic diseases.	405	53.3
Growth retardation often occurs in patients with inborn metabolic diseases during treatment.	231	31.1
Vitamin/mineral supplements should not be given to patients with inborn metabolic diseases.	132	17.4
Inborn metabolic diseases affect intelligence.	473	63.7
Inborn metabolic diseases can cause physical disability.	218	29.3
The treatment of inborn metabolic diseases requires medical nutrition therapy.	613	82.5
**Follow-up**		
There is no need for clinical follow-up after the diagnosis of inherited metabolic diseases.	117	15.4
Inborn metabolic diseases require lifelong follow-up.	615	82.8
Clinical follow-up of inborn metabolic diseases is carried out by Family Health Centers.	298	40.1
In inborn metabolic diseases, clinical follow-up should begin after 1 year of age.	477	62.7
Inborn metabolic diseases require a multidisciplinary team for clinical follow-up.	594	79.9

The distribution of health professional students by the scores they received from the questions measuring their level of knowledge about IMD is given in Table 4.

**Table 4:** The distribution of health professional students by the scores they received from the questions measuring their level of knowledge about IMD (n:761)

Variable	Min-Max value	X ± SD
Knowledge-level score for screening/diagnosis of IMD	1.0–10.0	3.93 ± 1.14
The knowledge-level score for treatment of IMD	2.0–11.0	7.39 ± 1.60
The knowledge-level score for follow-up of IMD	0.0–3.0	0.97 ± 0.86
Total score of IMD	6.0–20.0	14.23 ± 4.56

Factors affecting the knowledge level of healthcare professional students about IMD, encountering awareness-raising campaigns about IMD, the department, and grade of education are presented (Table 5).

It was shown that having consanguineous marriages in their parents and having the subject of IMD in their curriculum did not affect their level of knowledge.

It was determined that the knowledge-level score for screening/diagnosis of IMD, the knowledge-level score for follow-up, and the total knowledge score for IMD were higher (p < 0.05) in individuals who encountered an awareness-raising campaign about IMD than in individuals who did not (p < 0.05), whereas there was no difference between the knowledge-level scores for treatment (Table 5).

It was determined that the students in the nutrition and dietetics department had the highest IMD screening/diagnosis knowledge level, whereas the students in the audiology department had the lowest IMD screening/diagnosis knowledge score. Moreover, it was found that the students at the Department of Nutrition and Dietetics had the highest score in the treatment knowledge level, while the students at the Department of Social Work had the lowest score. In addition, in terms of follow-up knowledge level, it was determined that the students of the Department of Social Work had the highest score, while the students of the Department of Audiology had the lowest score. The highest total knowledge score of the IMD was found in the students of the Department of Nutrition and Dietetics, and the lowest score was found in the students of the Department of Child Development (Table 5).

**Table 5:** Factors affecting the knowledge level of healthcare professional students about IMD, encountering awareness-raising campaigns about IMD, the department, and grade of education

Variable	n	Knowledge-level score for screening/diagnosis of IMD X ± SD	p value	The knowledge-level score for treatment of IMD X ± SD	p value	The knowledge-level score for follow-up of IMD X ± SD	p value	Total score X ± SD	p value
**Consanguineous marriages in their families**			0.408		0.425		0.312		0.325
Yes	122	6.02 ± 1.33		7.26 ± 1.26		1.16 ± 0.83		13.25.±2.23	
No	639	5.71 ± 1.45		7.24 ± 1.55		0.65 ± 0.86		13.11 ± 2.31	
**Encountering awareness-raising campaigns about IMD**			0.015*		0.155		0.000*		0.050
Yes	127	5.08 ± 1.57		7.33 ± 1.47		1.10 ± 0.88		13.36 ± 2.39	
No	634	4.64 ± 1.24		7.17 ± 1.55		0.57 ± 0.34		13.11 ± 2.24	
**Department**			0.000*		0.002*		0.000*		0.000*
Nutrition and Dietetics	114	5.52 ± 1.47^c,e^		7.68 ± 1.19^a,c,e,g,ı^		1.62 ± 0.68^a,ı,j,l^		14.88 ± 2.21^e,g^	
Child Development	133	4.75 ± 1.55^j^		7.20 ± 1.56^ h,n^		1.36 ± 0.92^e,r^		12.60 ± 2.32^ L^	
Midwifery	90	4.88 ± 1.58^a,g^		7.25 ± 1.53^b,j^		0.90 ± 0.8^1b,c,d,f,g,n^		13.04 ± 2.24^a,c,ı^	
Occupational Therapy	117	5.46 ± 1.48^b,d,n^		7.18 ± 1.50^f,p^		1.36 ± 0.92^ h,k,t^		14.00 ± 2.59^d,f^	
Audiology	90	4.53 ± 1.57^,ı,o^		7.54 ± 1.23^ L,r^		0.62 ± 0.69^e,ö^		12.70 ± 2.07^j^	
Health Management	103	4.17 ± 1.15^ L^		7.22 ± 1.52^d^		0.90 ± 0.85^i,v^		12.80 ± 2.17^n^	
Social Work	114	5.16 ± 1.32^f,h,i,k,m^		6.83 ± 1.78^i,k,m,o^		1.67 ± 0.57^d,m,o,p,s,u,z^		13.98 ± 2.34^b,h,i,k,m,o^	
**Grade of education**			0.179		0.000*		0.475		0.233
1	102	4.69 ± 1.55		7.09 ± 1.13 ^a^		0.89 ± 0.68		12.12 ± 2.02	
2	343	4.24 ± 1.17		7.09 ± 1.63		1.03 ± 0.87		13.85 ± 2.29	
3	247	4.19 ± 1.73		8.64 ± 1.26 ^b^		0.99 ± 0.82		12.68 ± 2.18	
4	69	4.17 ± 1.19		7.04 ± 1.22		0.91 ± 0.84		12.19 ± 2.16	
**Having the subject of IMD in their curriculum.**			0.436		0.062		0.746		0.106
Yes	354	4.98 ± 1.56		7.17 ± 1.14		0.96 ± 0.15		13.32 ± 2.28	
No	407	4.89 ± 1.52		7.16 ± 1.17		0.98 ± 0.58		13.04 ± 2.32	

^a,b^ Different letters indicate significant difference between groups (p < 0.05)

## Discussion

It is aimed in the study; assessment of knowledge and awareness level of healthcare professional candidate students on inborn metabolic diseases. The level of exposure to awareness raising campaigns, education department and class level were determined as the factors affecting the knowledge level of health professionals.

Healthcare professionals play a key role in diagnosis, treatment, and follow-up [17]. However, it has been reported that they have difficulty providing support to these patients due to their insufficient level of knowledge about IMD [18]. It was determined that health sciences students were also inadequate in providing care to patients with IMD [15]. Moreover, recently, it has been highlighted that health sciences students have underestimated the epidemiology of IMD [19]. It has been shown that in one study, students believe that it is more important to prioritize common diseases in regard to health expenses, since IMDs affect a relatively small share of the population, as well as having an insufficient understanding of IMDs [20].

Increasing the level of knowledge and awareness of healthcare professionals working in the field of IMD is important in terms of public health as well as improving the effectiveness of healthcare services in general. Turkey is in a risky situation in terms of IMD due to consanguineous marriages, and providing early diagnosis and treatment services and ensuring follow-up is an extremely important issue that should not be overlooked. Hence, this study was planned and conducted to measure the level of knowledge of health sciences faculty students about IMD and to evaluate the factors affecting it [21].

In our study, when the IMD awareness status of individuals was evaluated, it was shown that more than half of them had heard of these diseases before, and the main source of information was social media. Social media is one of the most powerful communication and learning tools of the last period; through social networking sites, users can access a wide variety of information, including texts, videos, images, sounds, and the best professionals in their field [22]. It is well documented that almost all health sciences students (99.1%) use social media to support learning, which is associated with high academic achievement [23]. In another study evaluating the level of knowledge about IMD in health sciences students, similar to our findings, the majority of students reported that they used the internet to increase their knowledge about IMD [11]. It is predicted that implementing a formal curriculum for IMDs through verified and reliable websites or online classes will result in highly effective learning.

In our study, it was also found that the rate of individuals who had heard of rare disease organizations/platforms before was lower than the rate of having heard of IMDs. Compared to other countries, phenylketonuria, which is an IMD, is one of the highest incidence in Turkey [24, 25]. Newborns born in university hospitals in 27 cities in Turkey have been screened for phenylketonuria since 1986, and since 2006, a phenylketonuria newborn screening program has been implemented by the Ministry of Health throughout the country [10, 26]. When rare diseases are considered, awareness has increased recently thanks to patient associations around the world, and in 2019, some associations were united under the umbrella of the Rare Diseases Network, and the problems experienced started to be addressed [27]. Likewise, in our country, a parliamentary commission titled “Treatment and Care Methods for ALS (amyotrophic lateral sclerosis), SMA (spinal muscular atrophy), DMD (duchenne muscular dystrophy), MS (multiple sclerosis) and Other Diseases of Unknown Treatment and Detection of Diseases” was established in the Turkish Grand National Assembly in 2019 [28]. While there has been widespread awareness regarding the definition of rare diseases in the recent past, the term IMD is older, which is reflected in our results, as the names of rare disease organizations/platforms have been heard less frequently.

In our study, it was shown that few individuals encountered an awareness-raising campaign about IMD and that these individuals’ IMD screening/diagnosis, follow-up, and total knowledge scores were higher than those who did not. Although data measuring such effects on IMD have not yet been published, recent studies in the field of health have shown that campaigns result in an increased level of knowledge [29, 30]. In an awareness-raising campaign conducted in Italy on oral cancers in adolescents, it was found that such planning was appreciated among young students and provided effective results as a strategy to increase knowledge [29]. In an internet-based study on hearing loss involving Germany, Australia, Sweden, and the United Kingdom, increased awareness was demonstrated in online campaigns [30].

In our study, phenylketonuria (PKU), galactosemia, and glycogen storage diseases were identified as the top three most common IMDs. In a study conducted on health science students in Malaysia, biotinidase deficiency, phenylketonuria, and urea cycle diseases were ranked among the top three [11]. The prevalence of IMD varies among countries [24], which is thought to affect the frequency of hearing IMD. Since our country has the highest prevalence of PKU in the world, PKU has been stated as the most common disease.

When the knowledge levels of the individuals were evaluated, our findings were similar to the findings of recently published Saudi Arabia and Malaysia studies, concluding that both health and non-health sciences students lacked knowledge about the diagnosis and treatment of IMD [11, 14].

In a cross-sectional study by Alqrache et al.(14) in Saudi Arabia, it was shown that students in the fields of medicine and other health sciences did not have sufficient knowledge about IMD and its management, and the fact that IMD is a hereditary disease, its etiology, and clinical features were determined as the subjects on which the students had insufficient knowledge. In the study, the hereditary nature of these diseases and the importance of routine newborn screening programs and treatment were highlighted, and it was suggested that to increase awareness, education programs should be established [14].

It was also shown in this study that the most inadequate knowledge was about treatment, whereas knowledge about diagnosis and follow-up was found to be better. It was determined that in other studies [11, 14], the incidence and complications were evaluated in detail, but the treatment issue was not questioned.

In a study by Domaradzki et al. [15] with 350 medical students in Poland, 95.4% had inadequate or very poor knowledge about rare diseases, and 92.2% had inadequate or very poor knowledge about the care of their patients. In contrast, almost half (45.7%) believed that it was not necessary to include a course on rare diseases in the medical curriculum.

In a study by Vandeborne et al. [31] in Belgium, 86% of general practitioners and 72% of pediatricians had insufficient knowledge about rare diseases.

Only 40% of pediatricians in Australia, who were asked about their education and needs regarding rare diseases, stated that NHs adequately covered their undergraduate education and that they received information in their daily clinical practice through consultation with their colleagues, web-based resources, textbooks, and cell phone or tablet applications. stated [32].

Dharssi et al. [33], in their review of national rare disease policies of 11 countries, including Turkey, discussed the concepts of raising awareness, encouraging research, establishing specialized centers in the field, and strengthening patient organizations in their solution suggestions.

In our country, Bülbül et al. [34] evaluated the awareness of Fabry disease among medical doctors, showing that their level of knowledge was inadequate, and the lack of simple training methods and algorithms was suggested as the reason for inadequacy.

It has been shown that one out of every three IMD patients needs to wait more than two years to receive a correct diagnosis [35], and in another study, it was reported that 37% of patients with IMD were diagnosed after the age of 16 [36]. Not only pediatricians and family physicians but also many other branches, such as internal medicine, dermatology, physiotherapy and rehabilitation, orthopedics, and neurology, should raise awareness for individuals whose life expectancy is prolonged with early diagnosis and treatment. It is known that in many countries, there is no adult subspecialty training specific to IMDs [37].

In previous studies, it has been determined that female individuals have higher levels of knowledge about IMD due to their awareness of genetic tests and intense maternal instincts [14, 38, 39]. However, in this study, it was shown that although most of the individuals were women (97.7%), their level of knowledge was insufficient to a great extent, and it was determined that the factors of the department where they were educated and the situation of encountering awareness-raising campaigns were determinative.

In a study conducted in different fields, it was found that there was no difference between the knowledge levels of students in health sciences, dentistry, and pharmacy departments about IMD [11], but in our study, a difference was found between health sciences departments.

When the difference in terms of grade level was evaluated, the level of knowledge about treatment differed between the 1st and 3rd grades. In the third-grade curriculum, nutrition and dietetics students take 15 h of theoretical and practical coursework within the scope of the nutritional therapy in pediatric diseases course on IMDs, and it is possible to attribute the increased level of knowledge in the third grade to this experience.

In our study, however, it was shown that the inclusion of IMD in the curriculum did not affect knowledge levels. Only in the curriculum of the department of nutrition and dietetics are solutions on case examples of nutritional therapy and IMDs taught practically; in other departments, lectures on the subject are conducted theoretically for 4–6 hours in the total curriculum. It is generally discussed in the ‘nutrition’ course given by the faculty members of the nutrition and dietetics department within the compulsory course in the midwifery and child development program. In addition, IMD is covered in general terms in the “diseases in newborns” course in the midwifery and child development departments and in the “genetics” course on hearing problems in the audiology department. Since health management and social work programs are fields of social sciences, their curricula include the economic and social burden of diseases, whether they constitute a disability or not, and do not include disease issues specific to IMD.

It is thought that curriculum planning supported by practice, in which not only the subjects related to the department but also the basic issues about the IMD are handled in more detail, may be beneficial. Public health policies should focus on raising the level of awareness of this issue in all segments of society, as well as training health professionals to work in this field. To control inborn metabolic diseases, it is thought that integrating programs into the curricula of universities that train health professionals within the framework of competencies of the programs and increasing social awareness may be beneficial. In addition, there are also data indicating that students acquire knowledge about IMDs mostly out of interest or intellectual curiosity [15].

In the study by Farndon et al. [40], in which they evaluated genetic education and awareness, they determined that it would be important to explain its clinical significance before giving the information to physicians. In the evaluations made in Spain, it has been shown that it is necessary to implement actions aimed at collecting and disseminating existing information and resources on rare diseases, training the specialists providing primary healthcare services for diagnosis and referral to the appropriate center, and increasing the accessibility of the teaching staff dealing with children in this group to basic health information [41].

## Conclusions

The study revealed a need for immediate action to improve awareness of and knowledge about IMD among health science students in our country. Our findings are hoped to be a steppingstone toward increasing the number of studies to be conducted in other health and medical faculties in the future, as well as increasing the knowledge of future health professionals in the country regarding IMD. It will be of great value in future studies to conduct a more detailed analysis of factors influencing awareness and knowledge regarding IMD.

The treatment of IMD is performed by a multidisciplinary team in pediatric metabolism centers of children’s hospitals as an approach to acute metabolic deterioration and long-term treatment. The presence of health professionals such as a pediatric metabolism doctor, a dietitian specialized in IMD, a child development specialist, a physiotherapist/occupational therapist, a midwife/nurse, an audiologist, and a social worker in the team is crucial for effective treatment.

This is the first study to assess the level of knowledge and awareness of healthcare professionals such as dieticians, midwives, child development specialists, occupational therapists, audiologists, and social workers/health administrators who may play a role in the future treatment of IMD. There is a need for more comprehensive, multicentered studies in this field in the future, including physicians, pharmacists, physiotherapists, and nursing students. The tests used to measure the knowledge and awareness levels of future health professional students about inborn metabolic diseases are insufficient [12]. The effects of different test methods and the content of the education curriculum on the level of knowledge in faculties providing education in the field of health in international country examples were evaluated. However, there are differences between the tests and methods applied [42]. This study is the first to measure the knowledge level of inborn metabolic disease among health professional students in Turkey. Our study, which shows the data results in our country where the prevalence of IMD is known, will also raise awareness in international Health undergraduate education curriculum regulations in countries where IMD is observed frequently.

### Limitations of study

The study was conducted as a single center and could have been considered national data if it had been planned to include the faculties of health sciences of all universities. Despite these limitations, this is the first study to evaluate the level of knowledge and awareness of prospective health professionals in terms of IMD. There are few studies in the literature on patients, doctors, nurses, pharmacists, and physiotherapists regarding rare/genetic diseases, whereas there is no study that addresses dieticians, midwives, child development specialists, audiologists, occupational therapists, social workers, and health administrators who are included in the scope of this study. The role of future health professionals in the IMD care process is of great importance in controlling these diseases in Turkey. Furthermore, since this is a study conducted on health professionals, it will benefit the education of all women of childbearing age. It will shed light on the future in terms of revealing changes in the curriculum of universities that train different health professionals to improve IMD knowledge and awareness within the framework of their professional.

## Data Availability

The datasets generated and/or analysed during the current study are not publicly available due to privacy or ethical restrictions, but are available from the corresponding author on reasonable request.

## References

[1] Saudubray JM, Garcia-Cazorla À. Clinical approach to ınborn errors of metabolism In: JeanMarie Saudubray, Matthias R. Baumgartner John Walter Eds. Ed. Inborn Metabolic Diseases 6th edition. Springer Berlin, Heidelberg. 2016;3–69.

[2] Morava E, Rahman S, Peters V, Baumgartner MR, Patterson M, Zschocke J (2015). Quo vadis: the re-definition of “inborn metabolic diseases. J Inherit Metab Dis.

[3] Saudubray JM, Garcia-Cazorla À (2018). Inborn errors of metabolism overview: pathophysiology, manifestations, evaluation, and management. Pediat Clin N Am.

[4] Wertheim-Tysarowska K, Gos M, Sykut-Cegielska J, Bal J (2015). Genetic analysis in inherited metabolic disorders–from diagnosis to treatment. Own experience, current state of knowledge and perspectives. Dev Period Med.

[5] Ferreira CR, Rahman S, Keller M, Zschocke J, ICIMD Advisory Group (2021). An international classification of inherited metabolic disorders (ICIMD). J Inherit Metab Dis.

[6] Ramoser G, Caferri F, Radlinger B, Brunner-Krainz M, Herbst S, Huemer M, Hufgard-Leitner M, Kircher SG, Konstantopulou V, Löscher W (2022). 100 years of inherited metabolic disorders in Austria. A national registry of minimal birth prevalence, diagnosis, and clinical outcome of inborn errors of metabolism in Austria between 1921 and 2021. J Inherit Metab Dis.

[7] Dionisi-Vici C, Rizzo C, Burlina AB, Caruso U, Sabetta G, Uziel G, Abeni D (2002). Inborn errors of metabolism in the italian pediatric population: a national retrospective survey. J Pediatr.

[8] Afzal RM, Lund AM, Skovby F (2018). The impact of consanguinity on the frequency of inborn errors of metabolism. Mol Gen Metab Rep.

[9] Richter T, Nestler-Parr S, Babela R, Khan ZM, Tesoro T, Molsen E, Hughes DA, International Society for Pharmacoeconomics and Outcomes Research Rare Disease Special Interest Group (2015). Rare disease terminology and Definitions-A systematic global review: report of the ISPOR Rare Disease Special Interest Group. Value Health.

[10] Erçin S, Ovalı F (2019). Newborn screening. Klinik Tıp Pediatri Dergisi.

[11] Liew SH, Lim JY, Yahya HM, Rajikan R (2022). Knowledge and perception of inborn errors of metabolism (IEMs) among healthcare students at a selected public university in Klang Valley, Malaysia. Intract Rare Dis Res.

[12] Tebani A, Abily-Donval L, Afonso C, Marret S, Bekri S (2016). Clinical metabolomics: the new metabolic window for inborn errors of metabolism investigations in the post-genomic era. Inter J Mol Sci.

[13] Waters D, Adeloye D, Woolham D, Wastnedge E, Patel S, Rudan I (2018). Global birth prevalence and mortality from inborn errors of metabolism: a systematic analysis of the evidence. J Glob Health.

[14] Alqrache AT, Mostafa MM, Alqahtani MS, Atta HM (2020). Knowledge and awareness of metabolic inborn errors among male and female students at King Abdulaziz University – Rabigh. The Egypt J Med Educ.

[15] Walkowiak D, Domaradzki J (2020). Needs assessment study of rare diseases education for nurses and nursing students in Poland. Orphan J Rare Dis.

[16] George D, Mallery M. SPSS for Windows Step by Step: A Simple Guide and Reference, 17.0 update (10a ed.). 2010. Boston: Pearson.

[17] Agana M, Frueh J, Kamboj M, Patel DR, Kanungo S (2018). Common metabolic disorder (inborn errors of metabolism) concerns in primary care practice. Ann Trans Med.

[18] Tejada-Ortigosa EM, Flores-Rojas K, Moreno-Quintana L, Muñoz-Villanueva MC, Pérez-Navero JL, GilCampos M (2019). Necesidades sanitarias y socioeducativas de niños con enfermedades raras de tipo metabólico y sus familias: estudio cualitativo en un hospital de tercer nivel [Health and socio-educational needs of the families and children with rare metabolic diseases: qualitative study in a tertiary hospital]. Ann de Pediatr (Engl Ed).

[19] Jonas K, Waligóra M, Hołda M, Sulicka-Grodzicka J, Strach M, Podolec P, Kopeć G (2017). Knowledge on rare diseases among health care students – the effect of targeted education. Przeg Epidemiol.

[20] Ramalle-Gomara E, Ruiz E, Quinones C, Andres S, Iruzubieta J, Gil-de-Gomez J (2015). General knowledge and opinion of future health care and non-health care professionals on rare diseases. J Eval Clin Pract.

[21] Bozdoğan ST, Mungan HSO, Boga I, Yaşar HM, Büyükkurt S, Bişgin A (2021). The importance of genetic diagnosis for inherited metabolic Diseases: distribution and experience of Cukurova University Faculty of Medicine Balcali Hospital. ACU Sağlık Bilimleri Dergisi.

[22] Latif MZ, Hussain I, Saeed R, Qureshi MA, Maqsood U (2019). Use of smart phones and social media in medical education: trends, advantages, challenges and barriers. Acta Inf Med.

[23] Bich Diep P, Minh Phuong V, Dang Chinh N, Thi Hong Diem N, Bao Giang K. Health Science Students’ Use of Social Media for Educational Purposes: A Sample from a Medical University in Hanoi, Vietnam. Health Serv Insights. 2021;14:11786329211013549. 10.1177/11786329211013549. Erratum in: Health Serv Insight 2022;7(15):11786329211055319. 10.1177/11786329211013549 PMC8114251 34017181

[24] El-Metwally A, Yousef Al-Ahaidib L, Ayman Sunqurah A, Al-Surimi K, Househ M, Alshehri A, Da’ar OB, Abdul Razzak H, AlOdaib AN (2018). The prevalence of Phenylketonuria in Arab Countries, Turkey, and Iran: a systematic review. BioMed Res Int.

[25] Hillert A, Anikster Y, Belanger-Quintana A et al. The Genetic Landscape and Epidemiology of Phenylketonuria. Am J Hum Genet. 2020;6;107(2):234–250. 10.1016/j.ajhg.2020.06.006 PMC7413859 32668217

[26] Savli P, Ersoy M, Guner AE (2022). Evaluation of babies with hyperphenylalaninemia diagnosed in the National Newborn Screening Program in Istanbul in 2019. Int J Med Biochem.

[27] Güre MDP, İnce Ö (2021). Examining of Health Policy in Rare Diseases in terms of historical perspective in Turkey. J Int Health Sci Manag.

[28] Allotey PA, Allotey-Reidpath CD, Reidpath DD (2018). Health systems implications of rare genetic conditions in low-and middle-income countries: a case study approach. Crit Public Health.

[29] Rupel K, Ottaviani G, Gobbo M, Poropat A, Zoi V, Zacchigna S, Di Lenarda R, Biasotto M (2020). Campaign to increase awareness of oral Cancer risk factors among preadolescents. J Cancer Educ.

[30] D’Haese PSC, Van Rompaey V, De Bodt M, Van de Heyning P (2020). Can a Digital Awareness Campaign Change Knowledge and Beliefs regarding Cochlear Implants? A study in older adults in 5 european countries. Inquiry.

[31] Vandeborne L, van Overbeeke E, Dooms M (2019). Information needs of physicians regarding the diagnosis of rare diseases: a questionnaire-based study in Belgium. Orphan J Rare Dis.

[32] Zurynski Y, Gonzalez A, Deverell M (2017). Rare disease: a national survey of paediatricians’ experiences and needs. BMJ Paediatr Open.

[33] Dharssi S, Wong-Rieger D, Harold M, Terry S (2017). Review of 11 national policies for rare diseases in the context of key patient needs. Orphan J Rare Dis.

[34] Bulbul FS, Dursun O, Dursun ZE (2012). Physicians’, who are working in Kırıkkale, awareness of Fabry Disease and inherited metabolic Diseases. J LSD.

[35] Garau R (2016). The medical experience of a patient with a rare disease and her family. Orphan J Rare Dis.

[36] Gariani K, Nascimento M, Superti-Furga A (2020). Clouds over IMD? Perspectives for inherited metabolic diseases in adults from a retrospective cohort study in two swiss adult metabolic clinics. Orphan J Rare Dis.

[37] Hannah-Shmouni F, Stratakis CA, Sechi A, Langeveld M, Hiwot TG, Tchan MC, Mochel F, Lynd LD, Sirrs S (2019). Subspecialty training in adult inherited metabolic diseases: a call to action for unmet needs. Lancet Diabetes Endocrinol.

[38] Al-Enezi K, Mitra AK. (2017). Knowledge, Attitude, and Satisfaction of University Students Regarding Premarital Screening Programs in Kuwait. Eur J Environ Public Health 2017; 1(2): 07.

[39] Olwi D, Merdad L, Ramadan E (2016). Knowledge of genetics and attitudes toward genetic testing among college students in Saudi Arabia. Public Health Genom.

[40] Farndon PA, Bennett C (2008). Genetics education for health professionals: strategies and outcomes from a national initiative in the United Kingdom. J Genet Couns.

[41] Inés A, Cismondi R, Kohan H, Adams M, Bond R, Brown JD, Cooper et al. Guidelines for incorporating scientific knowledge and practice on rare diseases into higher education: neuronal ceroid lipofuscinoses as a model disorder, Biochimica et Biophysica Acta (BBA)- Molecular Basis of Disease Part B, 2015;1852(10)2316–2323, ISSN 0925–4439., C. Rodwell, S. Aymé, editors, 2014 Report on the State of the Art of Rare Disease Activities in Europe, 2014. 10.1016/j.bbadis.2015.06.018 26117801

[42] Kosan AMA, Toraman C (2020). Development and application of the commitment to Profession of Medicine Scale using classical test theory and item response theory. Croat Med J.

